# Probability of 5% or Greater Weight Loss or BMI Reduction to Healthy Weight Among Adults With Overweight or Obesity

**DOI:** 10.1001/jamanetworkopen.2023.27358

**Published:** 2023-08-07

**Authors:** Lyudmyla Kompaniyets, David S. Freedman, Brook Belay, Samantha L. Pierce, Emily M. Kraus, Heidi M. Blanck, Alyson B. Goodman

**Affiliations:** 1 Division of Nutrition, Physical Activity and Obesity, National Center for Chronic Disease Prevention and Health Promotion, Centers for Disease Control and Prevention, Atlanta, Georgia; 2 Public Health Informatics Institute, Taskforce for Global Health, Atlanta, Georgia; 3 Kraushold Consulting, Denver, Colorado

## Abstract

**Importance:**

Information on the probability of weight loss among US adults with overweight or obesity is limited.

**Objective:**

To assess the probability of 5% or greater weight loss, 10% or greater weight loss, body mass index (BMI) reduction to a lower BMI category, and BMI reduction to the healthy weight category among US adults with initial overweight or obesity overall and by sex and race.

**Design, Setting, and Participants:**

This cohort study obtained data from the IQVIA ambulatory electronic medical records database. The sample consists of US ambulatory patients 17 years or older with at least 3 years of BMI information from January 1, 2009, to February 28, 2022. Minimum age was set at 17 years to allow for the change in BMI or weight starting at 18 years. Maximum age was censored at 70 years.

**Exposures:**

Initial BMI (calculated as weight in kilograms divided by height in meters squared) category was the independent variable of interest, and the categories were as follows: lower than 18.5 (underweight), 18.5 to 24.9 (healthy weight), 25.0 to 29.9 (overweight), 30.0 to 34.9 (class 1 obesity), 35.0 to 39.9 (class 2 obesity), and 40.0 to 44.9 and 45.0 or higher (class 3 or severe obesity).

**Main Outcomes and Measures:**

The 2 main outcomes were 5% or greater weight loss (ie, a ≥5% reduction in initial weight) and BMI reduction to the healthy weight category (ie, BMI of 18.5-24.9).

**Results:**

The 18 461 623 individuals in the sample had a median (IQR) age of 54 (40-66) years and included 10 464 598 females (56.7%) as well as 7.7% Black and 72.3% White patients. Overall, 72.5% of patients had overweight or obesity at the initial visit. Among adults with overweight and obesity, the annual probability of 5% or greater weight loss was low (1 in 10) but increased with higher initial BMI (from 1 in 12 individuals with initial overweight to 1 in 6 individuals with initial BMI of 45 or higher). Annual probability of BMI reduction to the healthy weight category ranged from 1 in 19 individuals with initial overweight to 1 in 1667 individuals with initial BMI of 45 or higher. Both outcomes were generally more likely among females than males and were highest among White females. Over the 3 to 14 years of follow-up, 33.4% of persons with overweight and 41.8% of persons with obesity lost 5% or greater of their initial weight. At the same time, 23.2% of persons with overweight and 2.0% of persons with obesity reduced BMI to the healthy weight category.

**Conclusions and Relevance:**

Results of this cohort study indicate that the annual probability of 5% or greater weight loss was low (1 in 10) despite the known benefits of clinically meaningful weight loss, but 5% or greater weight loss was more likely than BMI reduction to the healthy weight category, especially for patients with the highest initial BMIs. Clinicians and public health efforts can focus on messaging and referrals to interventions that are aimed at clinically meaningful weight loss (ie, ≥5%) for adults at any level of excess weight.

## Key Points

**Question:**

What is the probability of health care–seeking adults with overweight or obesity observing a 5% or greater weight loss or body mass index (BMI) reduction to the healthy weight category?

**Findings:**

In this cohort study of 18 461 623 US patients with overweight and obesity, the annual probability of 5% or greater weight loss was low (1 in 10) but increased with higher initial BMI. The annual probability of reducing BMI to the healthy weight category was less likely, especially for individuals with initial BMI of 45 or higher.

**Meaning:**

Findings of this study suggest that clinicians and public health efforts can focus on messaging and referrals to interventions that support individuals with excess weight in achieving and sustaining meaningful weight loss.

## Teaser

This cohort study evaluates the changes over 3 to 14 years in body mass index and the differences in these changes by sex and race and ethnicity of US ambulatory patients.

## Introduction

Overweight and obesity together affect more than 70% of US adults^1^ and are risk factors for other chronic diseases, including type 2 diabetes,^2^ cardiovascular disease,^3^ and many cancers^4,5^ as well as severe illness from infectious diseases, such as COVID-19.^6,7^ Prior studies found that duration of obesity is an independent risk factor for the onset of chronic conditions, such as diabetes^2,8^ and cardiovascular disease.^3^ While the annual risk of developing a chronic condition among those with obesity is rather small, it compounds over time, suggesting that duration of obesity may affect the risk of chronic disease and mortality.^9,10^ At the same time, even modest (5%) weight loss at any body mass index (BMI; calculated as weight in kilograms divided by height in meters squared) category has been associated with improved health measures, including systolic and diastolic blood pressure, fasting glucose, hemoglobin A_1c_, and high-density lipoprotein cholesterol.^11,12,13^ A 5% reduction in body weight has been accepted as clinically meaningful,^14^ and a 5% to 10% goal for medically supervised weight loss was recommended by the 2013 guidelines for managing overweight and obesity.^15,16^ Understanding patterns of weight loss could help support populations, including Hispanic or Latino and non-Hispanic Black individuals, who are disproportionately affected by obesity due to factors, such as structural racism and race and ethnicity–based social and economic disadvantages.^17,18,19,20^

There is little information from large, longitudinal studies on BMI or body weight reduction among US adults with overweight or obesity and how the probability of weight reduction differs by sex or race and ethnicity. A prior study from the UK using ambulatory electronic medical record (AEMR) data showed that adults with obesity were unlikely to attain a healthy BMI but were more likely to attain a 5% or greater reduction in body weight.^21^ Prior US studies focused on patients with obesity and relied mostly on self-reported survey data^22^ or small randomized clinical trials for weight loss interventions.^23^ Thus, the present study used measured heights and weights from a large AEMR database to assess the probability of 5% or greater weight loss, 10% or greater weight loss, BMI reduction to a lower BMI category, and BMI reduction to the healthy weight category among US adults with initial overweight or obesity, overall and by sex and race.

## Methods

This analysis used the IQVIA AEMR database through the Observational Health Data Sciences and Informatics OMOP (Observational Medical Outcomes Partnership) common data model, version 5. Data were extracted using a software-as-a-service platform (E360; IQVIA). This activity was reviewed by the Centers for Disease Control and Prevention (CDC) and was conducted according to applicable US federal law and CDC policy. The CDC deemed this cohort study exempt from institutional review board approval because deidentified data were used. According to Privacy Analytics, which approved the Reidentification Risk Determination, informed consent was not required since the patients in the data asset were deidentified through IQVIA's proprietary encryption process. We followed the Strengthening the Reporting of Observational Studies in Epidemiology (STROBE) reporting guideline.^24^

### Study Population

The sample included ambulatory patients who were 17 years or older with at least 3 years of BMI information from January 1, 2009, to February 28, 2022 (eFigure in Supplement 1). A minimum age of 17 years was selected to allow for the change in BMI or weight starting at 18 years. Maximum age was censored at 70 years. Race and ethnicity (categorized as Asian, Black, Hispanic, White, other [no specific information available], or unknown) were reported by patients or clinicians and available as a single composite variable in the data set.

Height and weight measurements were cleaned using the growthcleanr (CRAN package; Comprehensive R Archive Network) algorithm for longitudinal cleaning of height and weight information in AEMR data sets.^25,26^ Heights, weights, and BMIs were also excluded in the following cases: height of less than 44 inches or more than 90 inches (to convert inches to centimeters, multiply by 2.54), weight of less than 55 pounds or more than 1000 pounds (to convert pounds to kilograms, multiply by 0.45), and BMI lower than 12 or higher than 110. One BMI per year per person was randomly selected. Individuals with pregnancy at any time during the study period were excluded.

### Measures and Outcomes

Initial BMI was the first BMI recorded in the database for each individual during the study period. Initial BMI category was the independent variable of interest and was classified as follows: lower than 18.5 (underweight), 18.5 to 24.9 (healthy weight), 25.0 to 29.9 (overweight), 30.0 to 34.9 (class 1 obesity), 35.0 to 39.9 (class 2 obesity), and 40.0 to 44.9 and 45.0 or higher as 2 categories of class 3 or severe obesity. Person-time was calculated as the number of days from initial BMI date to the outcome date (if an outcome occurred) or from initial BMI date to the last available BMI date within the study period (if no outcome occurred).

The 2 main outcomes were 5% or greater weight loss (ie, ≥5% reduction in initial weight) and BMI reduction to the healthy weight category (ie, BMI of 18.5-24.9). Two additional outcomes were assessed in supplemental analyses: 10% or greater weight loss and BMI reduction to a lower BMI category (eg, from class 1 obesity to overweight or lower).

### Statistical Analysis

We followed the methodological framework used by a prior UK study on this topic.^21^ We used frequencies and percentages to describe the patient sample. We evaluated the number of BMI records for each BMI category and calculated the number of records showing the following changes in BMI category: (1) 1 or more increases and no decreases, (2) 1 or more decreases and no increases, (3) no changes, and (4) both increases and decreases or weight cycling.

In a sample restricted to 13 381 050 persons with a BMI of 25 or higher (ie, overweight and obesity), we estimated several multivariable Poisson models with the outcomes of interest (≥5% weight loss or healthy weight) and person-years as exposure. Model 1 included the following covariates: 2-way interaction between initial BMI category and sex (including main effects) and initial age group (17-19, 20-29, 30-39, 40-49, 50-59, 60-69, or ≥70 years). Next, we used model 2 to assess the differences among Black and White males and females due to known disparities in BMI and BMI trajectories by race and sex among US adults.^27,28^ Model 2 was restricted to Black or White US adults, with the following covariates: initial age group and a 3-way interaction between initial BMI category, race (Black or White), and sex (including lower-order interactions and main effects). The other race and ethnicity categories were not included due to a large amount of missing data and small patient numbers.

Adjusted incidence rates of healthy weight per 10 000 person-years were obtained from the models, and mean annual probability of attaining an outcome from baseline was calculated as 1 − e^-r^ (where r was the adjusted incidence rate).^29^ Two-sided *P* < .05 was considered statistically significant. All analyses were conducted using R, version 4.2.1 and Stata, version 15.1 (StataCorp LLC).

Two supplemental analyses were also performed. The first supplemental analysis assessed 2 additional outcomes: 10% or greater weight loss and BMI reduction to a lower BMI category (eg, from BMI ≥45 to BMI of 40 to 44.9). The second supplemental analysis was performed in a subset of persons after excluding individuals with evidence of any of the following non–mutually exclusive potential reasons for intentional or unintentional weight loss at any time during the study period: eating disorders, antiobesity medication prescriptions, bariatric surgery, malignant neoplasm, metastatic solid tumor, Charlson Comorbidity Index of 3 or higher (indicating moderate or severe comorbidity),^30^ chemotherapy, and end-of-life or hospice care (eFigure in Supplement 1).

We assessed whether the 5% or greater weight loss was stable, which was defined in a prior study as having more weight loss than weight gain over time as quantified by a maximum weight gain of less than 5% from baseline and by the amount of maximum weight gain from baseline of less than 45% of the overall weight change magnitude (maximum-minimum) so that, overall, the pattern was still a weight loss.^31^ For patients who reduced BMI to the healthy weight category or to a lower BMI category, we evaluated whether these losses were reversed subsequently.

## Results

The 18 461 623 individuals in the sample had a median (IQR) age of 54 (40-66) years and included 10 464 598 females (56.7%) and 7 997 025 males (43.3%) as well as 7.7% Black and 72.3% White patients (Table 1). At the initial visit, 72.5% of individuals were in the overweight (BMI of 25.0-29.9) or obesity (BMI ≥30.0) category. Of these patients, 12.9% (12.7% males, 13.0% females) had only decreases in BMI category, 15.2% (14.1% males, 16.0% females) had only increases, 21.2% (20.4% males, 21.8% females) had weight cycling, and the rest had no BMI category changes (eTable 1 in Supplement 1; Table 2).

**Table 1.** Baseline Sample Characteristics From January 1, 2009, to February 28, 2022

Characteristic	Participants, No. (%) (N = 18 461 623)
Sex	
Male	7 997 025 (43.3)
Female	10 464 598 (56.7)
Initial age group, y^a^	
17-19	888 896 (4.8)
20-29	1 507 544 (8.2)
30-39	2 084 608 (11.3)
40-49	2 950 655 (16.0)
50-59	3 826 580 (20.7)
60-69	3 732 596 (20.2)
≥70	3 470 744 (18.8)
Race and ethnicity^b^	
Asian	385 937 (2.1)
Black	1 428 522 (7.7)
Hispanic	115 695 (0.6)
White	13 343 921 (72.3)
Other^c^	325 068 (1.8)
Unknown	2 862 480 (15.5)
US census region	
Northeast	3 653 471 (19.8)
Midwest	3 657 565 (19.8)
South	7 859 513 (42.6)
West	3 097 772 (16.8)
Unknown	193 302 (1.0)
Initial BMI category	
<18.5: Underweight	257 323 (1.4)
18.5-24.9: Healthy weight	4 823 250 (26.1)
25.0-29.9: Overweight	6 060 599 (32.8)
≥30.0: Obesity	7 320 451 (39.7)
30.0-34.9: Class 1 obesity	3 996 002 (21.6)
35.0-39.9: Class 2 obesity	1 916 406 (10.4)
40.0-44.9: Class 3 or severe obesity	842 423 (4.6)
≥45.0: Class 3 or severe obesity	565 620 (3.1)

Abbreviation: BMI, body mass index (calculated as weight in kilograms divided by height in meters squared).

^a^ Median (IQR) age: 54 (40-66) years.

^b^ Race and ethnicity data were reported by patients or clinicians and obtained from IQVIA ambulatory electronic medical record database.

^c^ No further information was available for other category.

**Table 2.** Number of BMI Records per Participant and Proportions Showing Changes in BMI Category From January 1, 2009, to February 28, 2022

Initial BMI category	Participants, No. (%)	Median (IQR)		Change in BMI category, No. (%)			
		Age, y	No. of BMI records	No changes, frequency	≥1 Decrease and no increases	≥1 Increase and no decreases	Both increases and decreases or weight cycling
**Males**							
Total	7 997 025 (100)	55 (41-67)	4 (3-6)	4 219 112 (52.8)	1 015 068 (12.7)	1 127 824 (14.1)	1 635 021 (20.4)
<18.5	61 294 (0.8)	25 (17-58)	4 (3-5)	23 438 (38.2)	NA	28 733 (46.9)	9123 (14.9)
18.5-24.9	1 583 376 (19.8)	52 (29-67)	4 (3-6)	997 523 (63.0)	29 079 (1.8)	338 856 (21.4)	217 918 (13.8)
25.0-29.9	3 083 494 (38.6)	57 (44-68)	5 (3-6)	1 780 218 (57.7)	337 203 (10.9)	398 335 (12.9)	567 738 (18.4)
≥30.0							
30.0-34.9	1 991 795 (24.9)	56 (45-66)	5 (3-7)	929 433 (46.7)	355 912 (17.9)	226 638 (11.4)	479 812 (24.1)
35.0-39.9	810 867 (10.1)	55 (43-64)	5 (3-7)	301 749 (37.2)	177 879 (21.9)	97 088 (12.0)	234 151 (28.9)
40.0-44.9	297 425 (3.7)	52 (41-62)	5 (3-7)	90 381 (30.4)	73 190 (24.6)	38 174 (12.8)	95 680 (32.2)
≥45.0	168 774 (2.1)	49 (39-59)	4 (3-6)	96 370 (57.1)	41 805 (24.8)	NA	30 599 (18.1)
**Females**							
Total	10 464 598 (100)	54 (39-66)	5 (3-6)	5 150 141 (49.2)	1 359 145 (13.0)	1 669 128 (16.0)	2 286 184 (21.8)
<18.5	196 029 (1.9)	46 (23-66)	4 (3-6)	85 178 (43.5)	NA	76 818 (39.2)	34 033 (17.4)
18.5-24.9	3 239 874 (31.0)	50 (32-65)	4 (3-6)	2 146 776 (66.3)	101 173 (3.1)	569 623 (17.6)	422 302 (13.0)
25.0-29.9	2 977 105 (28.4)	56 (42-68)	5 (3-7)	1 384 600 (46.5)	428 822 (14.4)	483 267 (16.2)	680 416 (22.9)
≥30.0							
30.0-34.9	2 004 207 (19.2)	56 (44-67)	5 (3-7)	781 936 (39.0)	372 159 (18.6)	300 752 (15.0)	549 360 (27.4)
35.0-39.9	1 105 539 (10.6)	54 (42-65)	5 (3-7)	363 648 (32.9)	236 758 (21.4)	159 754 (14.5)	345 379 (31.2)
40.0-44.9	544 998 (5.2)	53 (41-63)	5 (3-7)	152 971 (28.1)	131 387 (24.1)	78 914 (14.5)	181 726 (33.3)
≥45.0	396 846 (3.8)	50 (39-60)	5 (3-6)	235 032 (59.2)	88 846 (22.4)	NA	72 968 (18.4)

Abbreviations: BMI, body mass index (calculated as weight in kilograms divided by height in meters squared); NA, not applicable.

Over a maximum of 14 years of follow-up (January 1, 2009, to February 28, 2022), 33.4% of persons with initial overweight and 41.8% of persons with initial obesity BMI category had a 5% or greater reduction in weight (eTable 2 in Supplement 1). At the same time, 23.2% of persons with overweight and 2.0% of persons with obesity reduced BMI to the healthy weight (BMI of 18.5-24.9) category (eTable 3 in Supplement 1). The median (IQR) time was approximately 2.4 (1.4-4.0) years from initial visit to 5% or greater weight reduction and was 2.6 (1.5-4.4) years from initial visit to BMI reduction to the healthy weight category (eTable 4 in Supplement 1).

Among individuals with overweight or obesity, the adjusted annual probability of 5% or greater weight loss was 1 in 10 (Table 3). This probability increased with initial BMI category: from 1 in 12 individuals with initial overweight (1 in 14 males and 1 in 11 females) to 1 in 6 males and females with initial BMI of 45 or higher. Annual probability of a 5% or greater weight loss was slightly lower among Black females than White females (eg, 1 in 9 Black females compared with 1 in 8 White females with initial class 2 obesity [BMI of 35.0-39.9]).

**Table 3.** Probability of 5% or Greater Weight Loss Among Adults With Overweight and Obesity From January 1, 2009, to February 28, 2022

Initial BMI category	Total No.	Unadjusted No. with outcome over 3-14 y (%)	All, model 1^a^		By race, model 2^b^			
					White		Black	
			Adjusted incidence rate per 10 000 person-years (95% CI)	Annual probability	Adjusted incidence rate per 10 000 person-years (95% CI)	Annual probability	Adjusted incidence rate per 10 000 person-years (95% CI)	Annual probability
**All**								
Total	13 381 050	5 086 193 (38.0)	1007 (1006-1008)	1 in 10	NA	NA	NA	NA
25.0-29.9	6 060 599	2 023 712 (33.4)	840 (838-841)	1 in 12	NA	NA	NA	NA
30.0-34.9	3 996 002	1 548 089 (38.7)	1020 (1018-1021)	1 in 10	NA	NA	NA	NA
35.0-39.9	1 916 406	822 650 (42.9)	1199 (1196-1201)	1 in 9	NA	NA	NA	NA
40.0-44.9	842 423	392 469 (46.6)	1386 (1382-1391)	1 in 8	NA	NA	NA	NA
≥45.0	565 620	299 273 (52.9)	1719 (1712-1726)	1 in 6	NA	NA	NA	NA
**Males**								
Total	6 352 355	2 223 188 (35.0)	925 (924-926)	1 in 11	NA	NA	NA	NA
25.0-29.9	3 083 494	937 122 (30.4)	732 (731-734)	1 in 14	725 (723-726)	1 in 14	815 (809-822)	1 in 13
30.0-34.9	1 991 795	726 121 (36.5)	932 (930-934)	1 in 11	927 (925-930)	1 in 11	957 (948-965)	1 in 11
35.0-39.9	810 867	336 095 (41.4)	1146 (1142-1150)	1 in 9	1145 (1140-1149)	1 in 9	1122 (1108-1135)	1 in 9
40.0-44.9	297 425	135 316 (45.5)	1356 (1349-1363)	1 in 8	1361 (1353-1369)	1 in 8	1276 (1253-1299)	1 in 8
≥45.0	168 774	88 534 (52.5)	1713 (1702-1725)	1 in 6	1721 (1708-1735)	1 in 6	1600 (1568-1632)	1 in 7
**Females**								
Total	7 028 695	2 863 005 (40.7)	1088 (1087-1089)	1 in 10	NA	NA	NA	NA
25.0-29.9	2 977 105	1 086 590 (36.5)	937 (935-939)	1 in 11	940 (938-942)	1 in 11	912 (906-919)	1 in 11
30.0-34.9	2 004 207	821 968 (41.0)	1100 (1097-1102)	1 in 10	1108 (1105-1111)	1 in 10	1036 (1029-1044)	1 in 10
35.0-39.9	1 105 539	486 555 (44.0)	1246 (1243-1250)	1 in 9	1262 (1258-1266)	1 in 8	1158 (1149-1167)	1 in 9
40.0-44.9	544 998	257 153 (47.2)	1414 (1408-1419)	1 in 8	1438 (1432-1445)	1 in 7	1290 (1276-1304)	1 in 8
≥45.0	396 846	210 739 (53.1)	1724 (1717-1732)	1 in 6	1754 (1745-1763)	1 in 6	1598 (1582-1615)	1 in 7

Abbreviation: BMI, body mass index (calculated as weight in kilograms divided by height in meters squared); NA, not applicable.

^a^ Model 1 is a Poisson model on the sample of US adults with BMI of 25 or higher, with the outcome of a 5% or greater reduction in initial weight, exposure in person-years, and the following covariates: initial age group (17-19, 20-29, 30-39, 40-49, 50-59, 60-69, or ≥70 years) and a 2-way interaction between initial BMI category and sex (including main effects).

^b^ Model 2 is a Poisson model restricted to White or Black US adults with BMI of 25 or higher, with the outcome of a 5% or greater reduction in initial weight, exposure in person-years, and the following covariates: initial age group (17-19, 20-29, 30-39, 40-49, 50-59, 60-69, or ≥70 years) and a 3-way interaction between initial BMI category, race (Black or White), and sex (including lower-order interactions and main effects).

The adjusted annual probability of reducing BMI to the healthy weight category ranged from 1 in 19 individuals with initial overweight (1 in 23 males and 1 in 16 females) to 1 in 1667 individuals with initial BMI of 45 or higher (1 in 2870 males and 1 in 1201 females) (Table 4). This probability was higher among females than males and was the highest among White females (ranging from 1 in 16 with initial overweight to 1 in 1063 with initial BMI ≥45). Compared with White males, the annual probability among Black males was slightly higher after initial overweight (eg, 1 in 23 White males compared with 1 in 20 Black males) but lower after initial class 3 or severe obesity categories (eg, 1 in 2872 White males compared with 1 in 4179 Black males after initial BMI ≥45).

**Table 4.** Probability of Reducing BMI to Healthy Weight Category Among Adults With Overweight and Obesity From January 1, 2009, to February 28, 2022

Initial BMI category	Total No.	Unadjusted No. with outcome over 3-14 y (%)	All, model 1^a^		By race, model 2^b^			
					White		Black	
			Adjusted incidence rate per 10 000 person-years (95% CI)	Annual probability	Adjusted incidence rate per 10 000 person-years (95% CI)	Annual probability	Adjusted incidence rate per 10 000 person-years (95% CI)	Annual probability
**All**								
Total	13 381 050	1 548 339 (11.6)	273 (272-273)	1 in 37	NA	NA	NA	NA
25.0-29.9	6 060 599	1 403 821 (23.2)	541 (540-542)	1 in 19	NA	NA	NA	NA
30.0-34.9	3 996 002	119 291 (3.0)	65 (65-65)	1 in 154	NA	NA	NA	NA
35.0-39.9	1 916 406	18 854 (1.0)	21 (21-21)	1 in 479	NA	NA	NA	NA
40.0-44.9	842 423	4765 (0.6)	12 (11-12)	1 in 862	NA	NA	NA	NA
≥45.0	565 620	1608 (0.3)	6 (6–6)	1 in 1667	NA	NA	NA	NA
**Males**								
Total	6 352 355	654 321 (10.3)	221 (220-221)	1 in 46	NA	NA	NA	NA
25.0-29.9	3 083 494	608 613 (19.7)	447 (446-448)	1 in 23	437 (436-438)	1 in 23	500 (495-505)	1 in 20
30.0-34.9	1 991 795	39 854 (2.0)	42 (42-43)	1 in 236	42 (42-43)	1 in 237	50 (48-51)	1 in 201
35.0-39.9	810 867	4833 (0.6)	13 (13-14)	1 in 759	13 (13-13)	1 in 765	14 (12-15)	1 in 729
40.0-44.9	297 425	785 (0.3)	6 (6-7)	1 in 1635	6 (6-7)	1 in 1587	4 (3-6)	1 in 2253
≥45.0	168 774	236 (0.1)	3 (3-4)	1 in 2870	3 (3-4)	1 in 2872	2 (1-3)	1 in 4179
**Females**								
Total	7 028 695	894 018 (12.7)	325 (325-326)	1 in 31	NA	NA	NA	NA
25.0-29.9	2 977 105	795 208 (26.7)	640 (638-641)	1 in 16	643 (642-645)	1 in 16	553 (548-558)	1 in 19
30.0-34.9	2 004 207	79 437 (4.0)	84 (84-85)	1 in 119	88 (87-88)	1 in 115	73 (71-74)	1 in 138
35.0-39.9	1 105 539	14 021 (1.3)	28 (27-28)	1 in 359	29 (29-30)	1 in 341	21 (20-22)	1 in 474
40.0-44.9	544 998	3980 (0.7)	17 (16-17)	1 in 601	18 (17-19)	1 in 555	10 (9-11)	1 in 1008
≥45.0	396 846	1372 (0.3)	8 (8-9)	1 in 1201	9 (9-10)	1 in 1063	4 (3-5)	1 in 2526

Abbreviations: BMI, body mass index (calculated as weight in kilograms divided by height in meters squared); NA, not applicable.

^a^ Model 1 is a Poisson model on the sample of US adults with BMI of 25 or higher, with the outcome of healthy BMI, exposure in person-years, and the following covariates: initial age group (17-19, 20-29, 30-39, 40-49, 50-59, 60-69, or ≥70 years) and a 2-way interaction between initial BMI category and sex (including main effects).

^b^ Model 2 is a Poisson model restricted to White or Black US adults with BMI of 25 or higher, with the outcome of healthy weight, exposure in person-years, and the following covariates: initial age group (17-19, 20-29, 30-39, 40-49, 50-59, 60-69, or ≥70 years) and a 3-way interaction between initial BMI category, race (Black or White), and sex (including lower-order interactions and main effects).

The first supplemental analysis found that the annual probability of 10% or greater weight loss was 1 in 24 individuals, ranging from 1 in 32 individuals with initial overweight to 1 in 11 individuals with initial BMI of 45 or higher (eTable 5 in Supplement 1). The annual probability of reducing BMI to a lower category was 1 in 13 individuals, ranging from 1 in 19 individuals with initial overweight to 1 in 8 individuals with initial BMI of 45 or higher (eTable 6 in Supplement 1). The second supplemental analysis was performed in a subset of 11 119 541 individuals (83.1%) without documented reasons for intentional or unintentional weight loss. We found a slightly lower probability of 5% or greater weight loss in this subset (1 in 11), which ranged from 1 in 13 individuals with initial overweight (1 in 15 males and 1 in 12 females) to 1 in 7 males and females with initial BMI of 45 or higher (eTable 7 in Supplement 1). The probability of reducing BMI to the healthy weight category ranged from 1 in 20 individuals with initial overweight (1 in 23 males and 1 in 16 females) to 1 in 1612 individuals with BMI of 45 or higher (1 in 2197 males and 1 in 852 females).

We found that 76.5% of those with 5% or greater weight loss had a stable weight loss trajectory (ie, more weight loss than weight gain over time); this percentage increased with higher initial BMIs (eTable 2 in Supplement 1). At the same time, 42.8% of those who reduced BMI to the healthy weight category and 43.0% of those who moved to a lower BMI category reversed this change afterward (eTable 3 in Supplement 1).

## Discussion

In this cohort study, the annual probability of 5% or greater weight loss was low (1 in 10), ranging from 1 in 14 males and 1 in 11 females with overweight to 1 in 6 males and females with BMI of 45 or higher. At the same time, the probability of 5% or greater weight loss was considerably higher than that of reducing BMI to the healthy weight category, particularly for patients with higher initial BMIs. Over a maximum of 14 years of follow-up, 41.8% of patients with initial obesity had a 5% or greater reduction in weight, whereas only 2.0% of patients with initial obesity reduced BMI to the healthy weight category. Additionally, of those who had 5% or greater weight loss, 76.5% had a stable reduction, which might facilitate weight maintenance. Given the health benefits of clinically meaningful weight reduction at any level of excess weight,^11,12^ 5% or greater weight loss can be a reasonable target for obesity management efforts. Clinicians and public health efforts can focus on messaging and referrals to interventions that support adults with excess weight in achieving and sustaining clinically meaningful weight loss.

Narrowing the results to individuals with initial obesity, the annual probabilities of 5% or greater weight loss in this US study (eg, 1 in 11 males and 1 in 10 females with initial BMI of 30-34.9, or 1 in 6 males and females with initial BMI ≥45) were similar to those found in the prior UK cohort study by Fildes et al^21^ (eg, 1 in 12 males and 1 in 10 females with initial BMI of 30-34.9, or 1 in 5 males and 1 in 6 females with initial BMI ≥45). The annual probabilities of healthy weight were lower in the present study, especially among individuals with the highest initial BMIs (eg, 1 in 2870 males and 1 in 1201 females with initial BMI ≥45) compared with Fildes et al^21^ (eg, 1 in 362 males and 1 in 608 females with initial BMI ≥45). Furthermore, Fildes et al^21^ restricted the study to UK individuals with obesity, whereas this study also assessed these outcomes among US individuals with overweight.

Compared with males, females generally had a higher incidence of both outcomes, although the difference by sex in the incidence of 5% or greater weight loss narrowed with higher initial BMIs (not statistically significant at BMI ≥45). The findings of higher probability of weight loss among females were mostly consistent with prior clinical trials and survey studies.^32,33^ A prior study of the 2013 to 2016 National Health and Nutrition Examination Survey data showed that a higher percentage of females than males reported attempting to lose weight.^34^ Assessing differences by race, we found that the incidence of both outcomes was highest among White females (across all initial BMI categories) and lowest among White or Black males (depending on the initial BMI category).

In national data, the prevalence of obesity among adults 20 years or older increased from 35.7% in 2009 through 2010 to 42.4% in 2017 through 2018.^1^ Increasing prevalence of obesity, combined with the well-established health risks associated with obesity and the current finding of the low probability of reversing the pattern, signals a population-scale public health challenge in the US. These findings could, in part, be explained by barriers in availability of and access to obesity management options, including lifestyle interventions and pharmacotherapy.^35,36^ There is a continual need for policies and strategies that ensure community access to nutrition and physical activity opportunities.

This study focused on the probability of weight loss in a health care–seeking population with overweight or obesity regardless of any individual’s intention to lose weight. Several studies suggest that persons who are trying to lose weight may experience greater reductions in weight.^37,38,39,40^ For example, results of a 1-year randomized clinical trial among adults aged 18 to 75 years with a BMI of 25 to 45 showed that 42.8% of the treatment group enrolled in the commercial weight management program lost 5% of body weight compared with 24.7% of the control group using a do-it-yourself approach.^37^ Another trial on behavioral weight loss in a sample of 382 persons aged 18 to 25 years with a BMI of 25 to 45 found a 5% or greater weight reduction in 39.8% to 44.2% of the sample.^38^ Pharmacotherapy for obesity management has also demonstrated clinically meaningful weight loss.^39^ A recent review of the STEP (Semaglutide Treatment Effect in People With Obesity) program found that 86% to 89% of participants receiving semaglutide attained 5% or greater weight loss vs 29% to 48% of those receiving placebo.^40^

In this study, we did not differentiate between intentional and unintentional weight loss, both of which could have affected the patterns seen in this sample. A supplemental analysis of persons without documented reasons for intentional or unintentional weight loss found a slightly lower probability of 5% or greater weight loss compared with the main analysis. At the same time, the supplemental analysis found a higher probability of healthy weight in this subset vs the main sample among individuals with initial BMI of 45 or higher (eg, 1 in 852 females vs 1 in 1201 females). It is possible that the individuals with initial obesity who remained in the sample had better wellness or a greater ability to engage in healthy lifestyle behaviors, which facilitated weight reduction compared with individuals who were excluded from the sample. Further studies might consider assessing intentional or unintentional weight loss patterns at the population level as well as the probability of retaining the achieved weight or BMI reduction.

### Limitations

This study has limitations. First, we studied individuals with height and weight measured in a clinical setting; these results cannot be generalized to the broader population of US adults or those who do not seek health care. Second, our ability to analyze race and ethnicity data was limited because it was a single composite variable, there was no information on what constituted the other category of race and ethnicity, and 15.5% of patients had unknown race and ethnicity. We were also unable to verify how race and ethnicity information was collected; existing literature suggests that methods to ascertain and record race and ethnicity data within clinical settings are varied and suboptimal.^41^ Third, more than 60% of individuals in the IQVIA AEMR database had fewer than 3 years of BMI information (eFigure in Supplement 1). The obtained estimates might have been different if all individuals in the initial data set had 3 or more years of BMI information. Fourth, weight and height assessment in a clinical setting may be subject to several different measurement errors; however, we used the growthcleanr algorithm to detect, correct, and omit many of the erroneous values. Fifth, we were unable to control for important factors of weight loss, such as each individual’s participation in intensive health behavior and lifestyle interventions, level of physical activity, access to healthy and nutritious food, dietary intake, or socioeconomic determinants of health. Such information was not available in the database.

## Conclusions

In this cohort study of health care–seeking US adults with overweight or obesity, the annual probability of 5% or greater weight loss was low (1 in 10) despite the known benefits of clinically meaningful weight loss. At the same time, 5% or greater weight loss was more likely than BMI reduction to the healthy weight category, especially for individuals with the highest initial BMI. Clinicians and public health efforts can focus on messaging and referrals to interventions that are aimed at clinically meaningful weight loss (≥5%) for those at any level of excess weight.

## Notes

Supplement 1. **eFigure.** Sample Selection Process **eTable 1.** Number of BMI Records per Participant and Proportions Showing No Change, Increase, Decrease, or Weight Cycling Over 14 Years Following First BMI Record — IQVIA AEMR Database, United States, January 1, 2009 – February 28, 2022 **eTable 2.** Proportion of Individuals Who Had ≥5% Weight Loss and Those Who Had a Stable Weight Loss, By Initial BMI Category and Sex — IQVIA AEMR Database, United States, January 1, 2009 – February 28, 2022 **eTable 3.** Proportion of Individuals Who Reduced BMI to the Healthy Weight Category or a Lower BMI Category and Reversed That Reduction, by Initial BMI Category and Sex — IQVIA AEMR Database, United States, January 1, 2009 – February 28, 2022 **eTable 4.** Characteristics of Adults by Weight Loss Status — IQVIA AEMR Database, United States, January 1, 2009 – February 28, 2022 **eTable 5.** Supplemental Analysis (a): Annual Probability of ≥10% Weight Loss, by Sex, and Race — IQVIA AEMR Database, United States, January 1, 2009 – February 28, 2022 **eTable 6.** Supplemental Analysis (a): Annual Probability of Reducing BMI to a Lower Category: by Initial BMI Category, Sex, and Race — IQVIA AEMR Database, United States, January 1, 2009 – February 28, 2022 **eTable 7.** Supplemental Analysis (b): Adjusted Incidence and Adjusted Annual Probability of ≥5% Weight Loss and Reduction of BMI to the Healthy Weight Category in a Subset of Individuals Without Documented Causes of Intentional or Unintentional Weight Loss ^a^ — IQVIA AEMR Database, United States, January 1, 2009 – February 28, 2022

Supplement 2.
Data Sharing Statement
