# Errors in the Byline and Key Points

**DOI:** 10.1001/jamanetworkopen.2023.32740

**Published:** 2023-08-25

In the Original Investigation titled “Probability of 5% or Greater Weight Loss or BMI Reduction to Healthy Weight Among Adults With Overweight or Obesity”^1^ published August 7, 2023, one of Dr Belay’s academic degrees was omitted, and the number of patients listed in the Key Points was incorrect. Dr Belay’s name should be listed as Brook Belay, MD, MPH. The first sentence of the Findings section of the Key Points should be the following: “In this cohort study of 13 381 050 US patients with a BMI of 25 or higher, the annual probability of 5% or greater weight loss was low (1 in 10) but increased with higher initial BMI.” This article has been corrected.
