# Supplementary material for: Probability of 5% or Greater Weight Loss or BMI Reduction to Healthy Weight Among Adults With Overweight or Obesity
Source: JAMA Netw Open. 2023 Aug 7;6(8):e2327358. doi: 10.1001/jamanetworkopen.2023.27358 (PMC10407685; doi:10.1001/jamanetworkopen.2023.27358)
Supplement: Supplement 1. — eFigure. Sample Selection Process eTable 1. Number of BMI Records per Participant and Proportions Showing No Change, Increase, Decrease, or Weight Cycling Over 14 Years Following First BMI Record — IQVIA AEMR Database, United States, January 1, 2009 – February 28, 2022 eTable 2. Proportion of Individuals Who Had ≥5% Weight Loss and Those Who Had a Stable Weight Loss, By Initial BMI Category and Sex — IQVIA AEMR Database, United States, January 1, 2009 – February 28, 2022 eTable 3. Proportion of Individuals Who Reduced BMI to the Healthy Weight Category or a Lower BMI Category and Reversed That Reduction, by Initial BMI Category and Sex — IQVIA AEMR Database, United States, January 1, 2009 – February 28, 2022 eTable 4. Characteristics of Adults by Weight Loss Status — IQVIA AEMR Database, United States, January 1, 2009 – February 28, 2022 eTable 5. Supplemental Analysis (a): Annual Probability of ≥10% Weight Loss, by Sex, and Race — IQVIA AEMR Database, United States, January 1, 2009 – February 28, 2022 eTable 6. Supplemental Analysis (a): Annual Probability of Reducing BMI to a Lower Category: by Initial BMI Category, Sex, and Race — IQVIA AEMR Database, United States, January 1, 2009 – February 28, 2022 eTable 7. Supplemental Analysis (b): Adjusted Incidence and Adjusted Annual Probability of ≥5% Weight Loss and Reduction of BMI to the Healthy Weight Category in a Subset of Individuals Without Documented Causes of Intentional or Unintentional Weight Loss a — IQVIA AEMR Database, United States, January 1, 2009 – February 28, 2022 [file jamanetwopen-e2327358-s001.pdf]

Supplemental Online Content

Kompaniyets L, Freedman DS, Belay B, et al. Probability of 5% or greater weight loss or BMI reduction to healthy weight among adults with overweight or obesity. *JAMA Netw Open*. 2023;6(8):e2327358. doi:10.1001/jamanetworkopen.2023.27358

eFigure. Sample Selection Process

eTable 1. Number of BMI Records per Participant and Proportions Showing No Change, Increase, Decrease, or Weight Cycling Over 14 Years Following First BMI Record — IQVIA AEMR Database, United States, January 1, 2009 – February 28, 2022

eTable 2. Proportion of Individuals Who Had ≥5% Weight Loss and Those Who Had a Stable Weight Loss, By Initial BMI Category and Sex — IQVIA AEMR Database, United States, January 1, 2009 – February 28, 2022

eTable 3. Proportion of Individuals Who Reduced BMI to the Healthy Weight Category or a Lower BMI Category and Reversed That Reduction, by Initial BMI Category and Sex — IQVIA AEMR Database, United States, January 1, 2009 – February 28, 2022

eTable 4. Characteristics of Adults by Weight Loss Status — IQVIA AEMR Database, United States, January 1, 2009 – February 28, 2022

eTable 5. Supplemental Analysis (a): Annual Probability of ≥10% Weight Loss, by Sex, and Race — IQVIA AEMR Database, United States, January 1, 2009 – February 28, 2022

eTable 6. Supplemental Analysis (a): Annual Probability of Reducing BMI to a Lower Category: by Initial BMI Category, Sex, and Race — IQVIA AEMR Database, United States, January 1, 2009 – February 28, 2022

eTable 7. Supplemental Analysis (b): Adjusted Incidence and Adjusted Annual Probability of ≥5% Weight Loss and Reduction of BMI to the Healthy Weight Category in a Subset of Individuals Without Documented Causes of Intentional or Unintentional Weight Loss [a] — IQVIA AEMR Database, United States, January 1, 2009 – February 28, 2022

This supplemental material has been provided by the authors to give readers additional information about their work.

© 2023 Kompaniyets L et al. *JAMA Network Open.*

eFigure. Sample selection process

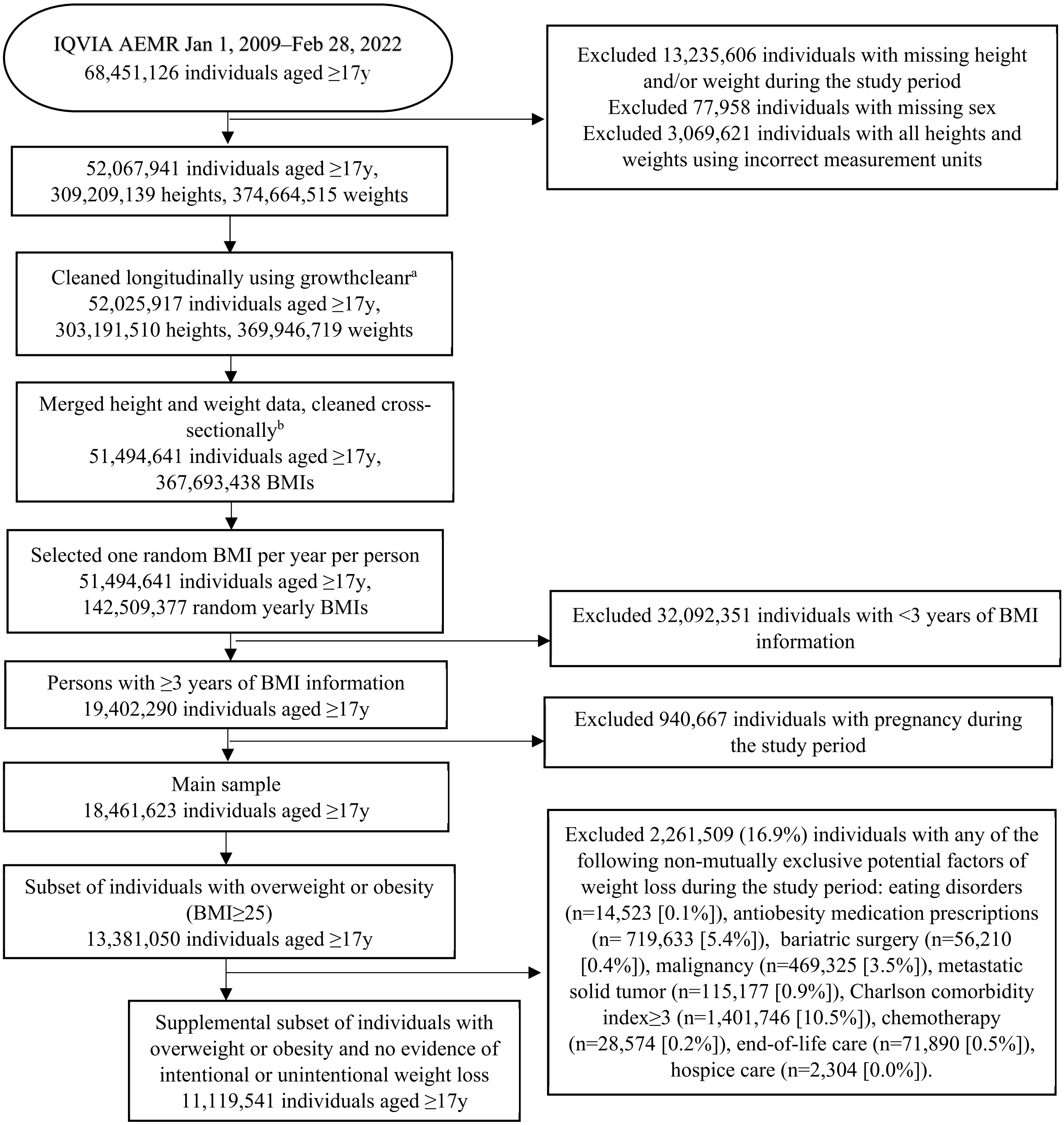

[a] growthcleanr is an R package to clean longitudinal height and weight measurements recorded in electronic health records. The growthcleanr algorithm compares each height and weight measurement for an individual to their other height and weight values to determine if that measurement is an error; it creates flags for erroneous values that can be corrected or removed by users. https://github.com/carriedaymont/growthcleanr

© 2023 Kompaniyets L et al. *JAMA Network Open.*

[b] Height and weight information was merged on event date; median height was used for BMI calculation. Heights, weights, and BMIs were excluded if they exceeded the following thresholds: height <44 inches [112 cm] or >90 inches [229 cm]; weight <25 kg [55 lbs] or >454 kg [1,000 lbs]; and BMI <12 kg/m^2 or >110 kg/m^2

© 2023 Kompaniyets L et al. *JAMA Network Open.*

eTable 1. Number of BMI Records per Participant and Proportions Showing No Change, Increase, Decrease, or Weight Cycling Over 14 Years Following First BMI Record — IQVIA AEMR database, United States, January 1, 2009 – February 28, 2022

**Initial BMI category**	N (Col %)	Median (IQR)		N (Row %)			
		Age, years	Number of BMI records	All Records Show No Change in BMI Category, Frequency	≥ 1 Decrease in BMI Category and No Increases	≥ 1 Increase in BMI Category and No Decreases	Records Show Both Increases and Decreases in BMI Category
**Total**	18,461,623 (100.0)	54 (40-66)	4 (3-6)	9,369,253 (50.7)	2,374,213 (12.9)	2,796,952 (15.2)	3,921,205 (21.2)
<18.5	257,323 (1.4)	42 (20-65)	4 (3-6)	108,616 (42.2)	NA	105,551 (41.0)	43,156 (16.8)
18.5–24.9	4,823,250 (26.1)	50 (31-66)	4 (3-6)	3,144,299 (65.2)	130,252 (2.7)	908,479 (18.8)	640,220 (13.3)
25.0–29.9	6,060,599 (32.8)	57 (43-68)	5 (3-6)	3,164,818 (52.2)	766,025 (12.6)	881,602 (14.5)	1,248,154 (20.6)
30.0–34.9	3,996,002 (21.6)	56 (44-67)	5 (3-7)	1,711,369 (42.8)	728,071 (18.2)	527,390 (13.2)	1,029,172 (25.8)
35.0–39.9	1,916,406 (10.4)	54 (43-65)	5 (3-7)	665,397 (34.7)	414,637 (21.6)	256,842 (13.4)	579,530 (30.2)
40.0–44.9	842,423 (4.6)	53 (41-62)	5 (3-7)	243,352 (28.9)	204,577 (24.3)	117,088 (13.9)	277,406 (32.9)
≥45.0	565,620 (3.1)	50 (39-60)	5 (3-6)	331,402 (58.6)	130,651 (23.1)	NA	103,567 (18.3)

© 2023 Kompaniyets L et al. *JAMA Network Open.*

eTable 2. Proportion of Individuals Who Had ≥5% Weight Loss and Those Who Had a Stable Weight Loss, By Initial BMI Category and Sex — IQVIA AEMR Database, United States, January 1, 2009 – February 28, 2022

Initial BMI category (kg/m^2)	Total N	Had ≥5% weight loss n (% of Total N)	Had stable ≥5% weight loss[a] n (% of Total N)	Stable 5% weight loss (% of n with a ≥5% weight loss)
**All**	13,381,050	5,086,193 (38.0)	3,893,015 (29.1)	76.5
25.0–29.9	6,060,599	2,023,712 (33.4)	1,530,938 (25.3)	75.6
≥30.0	7,320,451	3,062,481 (41.8)	2,362,077 (32.3)	77.1
30.0–34.9	3,996,002	1,548,089 (38.7)	1,185,818 (29.7)	76.6
35.0–39.9	1,916,406	822,650 (42.9)	631,227 (32.9)	76.7
40.0–44.9	842,423	392,469 (46.6)	304,007 (36.1)	77.5
≥45.0	565,620	299,273 (52.9)	241,025 (42.6)	80.5
**Males**	6,352,355	2,223,188 (35.0)	1,756,185 (27.6)	79.0
25.0–29.9	3,083,494	937,122 (30.4)	738,049 (23.9)	78.8
≥30.0	3,268,861	1,286,066 (39.3)	1,018,136 (31.1)	79.2
30.0–34.9	1,991,795	726,121 (36.5)	573,253 (28.8)	78.9
35.0–39.9	810,867	336,095 (41.4)	264,775 (32.7)	78.8
40.0–44.9	297,425	135,316 (45.5)	106,731 (35.9)	78.9
≥45.0	168,774	88,534 (52.5)	73,377 (43.5)	82.9
**Females**	7,028,695	2,863,005 (40.7)	2,136,830 (30.4)	74.6
25.0–29.9	2,977,105	1,086,590 (36.5)	792,889 (26.6)	73.0
≥30.0	4,051,590	1,776,415 (43.8)	1,343,941 (33.2)	75.7
30.0–34.9	2,004,207	821,968 (41.0)	612,565 (30.6)	74.5
35.0–39.9	1,105,539	486,555 (44.0)	366,452 (33.1)	75.3
40.0–44.9	544,998	257,153 (47.2)	197,276 (36.2)	76.7
≥45.0	396,846	210,739 (53.1)	167,648 (42.2)	79.6

[a] Defined by: (1) a net weight loss of > 0 kg over the measured period; and (2) the maximum weight loss from baseline was ≥ 5%; and (3) the individual had more weight loss than weight gain over time, and this was quantified by (a) a maximum weight gain of < 5% from baseline, or (b) the amount of maximum weight gain from baseline was < 45% of the overall weight change magnitude (maximum - minimum), so that overall the trend is still a weight loss.

© 2023 Kompaniyets L et al. *JAMA Network Open.*

eTable 3. Proportion of Individuals Who Reduced BMI to the Healthy Weight Category Or a Lower BMI Category and Reversed that Reduction, by Initial BMI Category and Sex — IQVIA AEMR Database, United States, January 1, 2009 – February 28, 2022

**Initial BMI category (kg/m^2)**	**Total N**	**Healthy BMI category**			**Lower BMI category**		
		**Attained n (row %)**	**Reversed n (row %)**	**Reversed (% of attained)**	**Attained n (row %)**	**Reversed n (row %)**	**Reversed (% of attained)**
**All**	13,381,050	1,548,339 (11.6)	662,863 (5.0)	42.8	4,245,418 (31.7)	1,827,616 (13.7)	43.0
25.0–29.9	6,060,599	1,403,821 (23.2)	610,617 (10.1)	43.5	1,406,120 (23.2)	612,038 (10.1)	43.5
≥30.0	7,320,451	144,518 (2.0)	52,246 (0.7)	36.2	2,839,298 (38.8)	1,215,578 (16.6)	42.8
30.0–34.9	3,996,002	119,291 (3.0)	42,719 (1.1)	35.8	1,382,118 (34.6)	602,650 (15.1)	43.6
35.0–39.9	1,916,406	18,854 (1.0)	6,956 (0.4)	36.9	812,917 (42.4)	349,705 (18.2)	43.0
40.0–44.9	842,423	4,765 (0.6)	1,883 (0.2)	39.5	410,045 (48.7)	172,027 (20.4)	42.0
≥45.0	565,620	1,608 (0.3)	688 (0.1)	42.8	234,218 (41.4)	91,196 (16.1)	38.9
**Males**	6,352,355	654,321 (10.3)	278,277 (4.4)	42.5	1,831,776 (28.8)	788,980 (12.4)	43.1
25.0–29.9	3,083,494	608,613 (19.7)	262,646 (8.5)	43.2	609,799 (19.8)	263,344 (8.5)	43.2
≥30.0	3,268,861	45,708 (1.4)	15,631 (0.5)	34.2	1,221,977 (37.4)	525,636 (16.1)	43.0
30.0–34.9	1,991,795	39,854 (2.0)	13,461 (0.7)	33.8	664,515 (33.4)	290,650 (14.6)	43.7
35.0–39.9	810,867	4,833 (0.6)	1,765 (0.2)	36.5	341,140 (42.1)	147,215 (18.2)	43.2
40.0–44.9	297,425	785 (0.3)	309 (0.1)	39.4	143,918 (48.4)	60,560 (20.4)	42.1
≥45.0	168,774	236 (0.1)	96 (0.1)	40.7	72,404 (42.9)	27,211 (16.1)	37.6
**Females**	7,028,695	894,018 (12.7)	384,586 (5.5)	43.0	2,413,642 (34.3)	1,038,636 (14.8)	43.0
25.0–29.9	2,977,105	795,208 (26.7)	347,971 (11.7)	43.8	796,321 (26.7)	348,694 (11.7)	43.8
≥30.0	4,051,590	98,810 (2.4)	36,615 (0.9)	37.1	1,617,321 (39.9)	689,942 (17.0)	42.7
30.0–34.9	2,004,207	79,437 (4.0)	29,258 (1.5)	36.8	717,603 (35.8)	312,000 (15.6)	43.5
35.0–39.9	1,105,539	14,021 (1.3)	5,191 (0.5)	37.0	471,777 (42.7)	202,490 (18.3)	42.9
40.0–44.9	544,998	3,980 (0.7)	1,574 (0.3)	39.5	266,127 (48.8)	111,467 (20.5)	41.9
≥45.0	396,846	1,372 (0.3)	592 (0.1)	43.1	161,814 (40.8)	63,985 (16.1)	39.5

© 2023 Kompaniyets L et al. *JAMA Network Open.*

eTable 4. Characteristics of Adults by Weight Loss Status — IQVIA AEMR database, United States, January 1, 2009 – February 28, 2022

	N (%)			
**Characteristics**	Lost ≥5% weight	Did not lose ≥5% weight	Reduced BMI to the healthy weight category	Did not reduce BMI to the healthy weight category
**Total**	5,086,193 (100.0)	8,294,857 (100.0)	1,548,339 (100.0)	11,832,711 (100.0)
**Age at initial year: mean / median (IQR)**	55 / 58 (46-69)	52 / 54 (41-64)	56 / 61 (46-70)	53 / 55 (43-66)
**Bariatric surgery ever** [a]				
Yes	31,677 (0.6)	24,533 (0.3)	4,680 (0.3)	51,530 (0.4)
No	5,054,516 (99.4)	8,270,324 (99.7)	1,543,659 (99.7)	11,781,181 (99.6)
**Antiobesity medication prescriptions ever** [b]				
Yes	330,478 (6.5)	389,155 (4.7)	35,574 (2.3)	684,059 (5.8)
No	4,755,715 (93.5)	7,905,702 (95.3)	1,512,765 (97.7)	11,148,652 (94.2)
**Charlson comorbidity index** [c]				
Meanat initial year	0.6	0.5	0.6	0.5
0	3,322,630 (65.3)	6,076,840 (73.3)	1,076,045 (69.5)	8,323,425 (70.3)
1	1,030,376 (20.3)	1,389,583 (16.8)	263,672 (17.0)	2,156,287 (18.2)
2	391,041 (7.7)	465,855 (5.6)	114,186 (7.4)	742,710 (6.3)
≥3	342,146 (6.7)	362,579 (4.4)	94,436 (6.1)	610,289 (5.2)
**Years from first BMI to weight/BMI reduction**				
Mean / Median (IQR)	3.0/ 2.4 (1.4-4.0)		3.2/2.6 (1.5-4.4)	

[a] Bariatric surgery was assessed at any time during January 1, 2008–February 28, 2022, and was defined using CPT codes (43644, 43645, 43659, 43770, 43842,43843,43485–43847, S2082, S2085).

[b] Antiobesity medication prescriptions were defined using RxNorm and NDC codes at any time during January 1, 2008–February 28, 2022, and included liraglutide, semaglutide, diethylpropion, orlistat, phendimetrazine, benzphetamine, phentermine, phentermine-topiramate, setmelanotide, and naltrexone/bupropion.

[c] Charlson comorbidity index was defined using SNOMED codes.

© 2023 Kompaniyets L et al. *JAMA Network Open.*

eTable 5. Supplemental Analysis (a): Annual Probability of ≥10% Weight Loss, by Sex, and Race — IQVIA AEMR database, United States, January 1, 2009 – February 28, 2022

**Initial BMI category (kg/m²)**	Total N	Unadjusted N (row %) with outcome over 3-14 years	**All (Model 1)[a]**		**By race (Model 2)[a]**			
					**White**		**Black**	
			Adjusted incidence rate (95% CI), per 10,000 person-years	Annual probabilit y	Adjusted incidence rate (95% CI), per 10,000 person-years	Annual probability	Adjusted incidence rate (95% CI), per 10,000 person-years	Annual probability
**All**								
Total	13,381,050	2,474,903 (18.5)	429 (428–429)	1 in 24				
25.0-29.9	6,060,599	875,119 (14.4)	321 (320–322)	1 in 32				
30.0-34.9	3,996,002	747,600 (18.7)	430 (429–431)	1 in 24				
35.0-39.9	1,916,406	437,617 (22.8)	550 (548–552)	1 in 19				
40.0-44.9	842,423	225,271 (26.7)	684 (681–687)	1 in 15				
≥45.0	565,620	189,296 (33.5)	944 (940–949)	1 in 11				
Total	6,352,355	969,547 (15.3)	364 (363–365)	1 in 28				
25.0-29.9	3,083,494	354,117 (11.5)	245 (244-246)	1 in 41	245 (244-246)	1 in 41	301 (297-304)	1 in 34
30.0-34.9	1,991,795	318,363 (16.0)	358 (357-359)	1 in 28	360 (358-361)	1 in 28	368 (363-372)	1 in 28
35.0-39.9	810,867	167,182 (20.6)	496 (493-498)	1 in 21	499 (496-502)	1 in 21	471 (463-479)	1 in 22
40.0-44.9	297,425	74,372 (25.0)	647 (642-652)	1 in 16	655 (650-660)	1 in 16	582 (568-597)	1 in 18
≥45.0	168,774	55,513 (32.9)	941 (933-949)	1 in 11	947 (937-956)	1 in 11	878 (855-900)	1 in 12
Total	7,028,695	1,505,356 (21.4)	491 (490–492)	1 in 21				
25.0-29.9	2,977,105	521,002 (17.5)	390 (389-391)	1 in 26	398 (396-399)	1 in 26	377 (373-381)	1 in 27
30.0-34.9	2,004,207	429,237 (21.4)	495 (494-497)	1 in 21	508 (506-510)	1 in 20	440 (436-444)	1 in 23
35.0-39.9	1,105,539	270,435 (24.5)	599 (597-601)	1 in 17	618 (616-621)	1 in 17	515 (509-521)	1 in 20
40.0-44.9	544,998	150,899 (27.7)	717 (713-720)	1 in 14	742 (738-747)	1 in 14	604 (595-613)	1 in 17
≥45.0	396,846	133,783 (33.7)	947 (942-952)	1 in 11	978 (972-984)	1 in 11	826 (815-838)	1 in 13

[a] Model 1 is a Poisson model on the sample of US adults with BMI≥25, with the outcome of reducing weight by 10%, exposure in person-years, and the following covariates: two-way interaction between initial BMI category and sex, including main effects, and initial age group (17–19, 20–29, 30–39, 40–49, 50–59, 60–69, 70+). Model 2 is a Poisson model restricted to White or Black US adults with BMI ≥25, with the outcome of reducing weight by 10%,, exposure in person-years, and the following covariates: three-way interaction between initial BMI category, race (Black or White) and sex, including lower-order interactions and main effects, and initial age group (17–19, 20–29, 30–39, 40–49, 50–59, 60–69, 70+).

© 2023 Kompaniyets L et al. *JAMA Network Open.*

eTable 6. Supplemental analysis (a): Annual Probability of Reducing BMI to a Lower Category: by Initial BMI Category, Sex, and Race — IQVIA AEMR database, United States, January 1, 2009 – February 28, 2022

**Initial BMI category (kg/m^2)**	Total N	Unadjusted N (row %) with outcome over 3-14 years	**All (Model 1)[a]**		**By race (Model 2)[a]**			
					**White**		**Black**	
			Adjusted incidence rate (95% CI), per 10,000 person-years	Annual probability	Adjusted incidence rate (95% CI), per 10,000 person-years	Annual probability	Adjusted incidence rate (95% CI), per 10,000 person-years	Annual probability
**Both sexes**								
Total	13,381,050	4,245,418 (31.7)	798 (797-799)	1 in 13				
25.0-29.9	6,060,599	1,406,120 (23.2)	543 (542-544)	1 in 19				
30.0-34.9	3,996,002	1,382,118 (34.6)	862 (860-863)	1 in 12				
35.0-39.9	1,916,406	812,917 (42.4)	1,144 (1,142-1,147)	1 in 9				
40.0-44.9	842,423	410,045 (48.7)	1,417 (1,412-1,421)	1 in 8				
≥45.0	565,620	234,218 (41.4)	1,260 (1,254-1,265)	1 in 8				
**Males**								
Total	6,352,355	1,831,776 (28.8)	739 (738–740)	1 in 14				
25.0-29.9	3,083,494	609,799 (19.8)	449 (448-450)	1 in 23	438 (437-440)	1 in 23	499 (494-503)	1 in 21
30.0-34.9	1,991,795	664,515 (33.4)	849 (847-851)	1 in 12	840 (838-843)	1 in 12	846 (839-854)	1 in 12
35.0-39.9	810,867	341,140 (42.1)	1,196 (1,192-1,200)	1 in 9	1,190 (1,186-1,195)	1 in 9	1,154 (1,140-1,168)	1 in 9
40.0-44.9	297,425	143,918 (48.4)	1,514 (1,506-1,521)	1 in 7	1,515 (1,506-1,524)	1 in 7	1,434 (1,409-1,459)	1 in 7
≥45.0	168,774	72,404 (42.9)	1,352 (1,342-1,362)	1 in 8	1,373 (1,361-1,385)	1 in 8	1,167 (1,140-1,194)	1 in 9
**Females**								
Total	7,028,695	2,413,642 (34.3)	859 (858–860)	1 in 12				
25.0-29.9	2,977,105	796,321 (26.7)	643 (641-644)	1 in 16	647 (645-648)	1 in 16	555 (550-560)	1 in 19
30.0-34.9	2,004,207	717,603 (35.8)	933 (931-935)	1 in 11	940 (938-943)	1 in 11	838 (831-844)	1 in 12
35.0-39.9	1,105,539	471,777 (42.7)	1,216 (1,213-1,220)	1 in 9	1,228 (1,224-1,232)	1 in 9	1,108 (1,099-1,118)	1 in 10
40.0-44.9	544,998	266,127 (48.8)	1,513 (1,507-1,519)	1 in 7	1,534 (1,527-1,541)	1 in 7	1,379 (1,364-1,393)	1 in 8
≥45.0	396,846	161,814 (40.8)	1,237 (1,231-1,243)	1 in 9	1,278 (1,270-1,285)	1 in 8	1,049 (1,035-1,062)	1 in 10

[a] Model 1 is a Poisson model on the sample of US adults with BMI≥25, with the outcome of reducing BMI to a lower category, exposure in person-years, and the following covariates: two-way interaction between initial BMI category and sex, including main effects, and initial age group (17–19, 20–29, 30–39, 40–49, 50–59, 60–69, 70+). Model 2 is a Poisson model restricted to White or Black US adults with BMI ≥25, with the outcome of reducing BMI to a lower category, exposure in person-years, and the following covariates: three-way interaction between initial BMI category, race (Black or White) and sex, including lower-order interactions and main effects, and initial age group (17–19, 20–29, 30–39, 40–49, 50–59, 60–69, 70+).

© 2023 Kompaniyets L et al. *JAMA Network Open.*

eTable 7. Supplemental Analysis (b): Adjusted Incidence and Adjusted Annual Probability of ≥5% Weight Loss and Reduction of BMI to the Healthy Weight Category in a Subset of Individuals without Documented Causes of Intentional or Unintentional Weight Loss[a] — IQVIA AEMR database, United States, January 1, 2009 – February 28, 2022

**Initial BMI category (kg/m^2)**	**Total N**	**Outcome: ≥5% reduction in initial weight (Model 1)**			**Outcome: healthy weight (Model 1)**		
		N (row %) with outcome	Adjusted incidence rate (95% CI), per 10,000 person-years	Annual probability	N (row %) with outcome	Adjusted incidence rate (95% CI), per 10,000 person-years	Annual probability
**Both sexes**							
**Total**	11,119,541	3,986,885 (35.9)	944 (943-945)	1 in 11	1,279,383 (11.5)	271 (270-271)	1 in 37
25.0–29.9	5,269,730	1,679,072 (31.9)	799 (798-800)	1 in 13	1,172,486 (22.2)	520 (520-521)	1 in 20
30.0–34.9	3,308,562	1,217,725 (36.8)	967 (965-969)	1 in 11	88,376 (2.7)	59 (59-59)	1 in 170
35.0–39.9	1,503,904	610,212 (40.6)	1,130 (1,127-1,133)	1 in 9	13,770 (0.9)	20 (19-20)	1 in 508
40.0–44.9	633,011	277,885 (43.9)	1,302 (1,296-1,307)	1 in 8	3,570 (0.6)	12 (11-12)	1 in 857
≥45.0	404,334	201,991 (50.0)	1,614 (1,606-1,621)	1 in 7	1,181 (0.3)	6 (6-7)	1 in 1,612
**Males**							
**Total**	5,337,394	1,748,583 (32.8)	860 (859-862)	1 in 12	531,885 (10.0)	215 (215-216)	1 in 47
25.0–29.9	2,684,923	768,489 (28.6)	688 (687-690)	1 in 15	499,115 (18.6)	448 (446-449)	1 in 23
30.0–34.9	1,667,292	574,196 (34.4)	880 (878-883)	1 in 12	28,537 (1.7)	44 (44-45)	1 in 226
35.0–39.9	640,718	250,342 (39.1)	1,082 (1,077-1,086)	1 in 10	3,509 (0.5)	15 (15-16)	1 in 649
40.0–44.9	223,683	95,641 (42.8)	1,274 (1,266-1,282)	1 in 8	558 (0.2)	8 (7-8)	1 in 1,323
≥45.0	120,778	59,915 (49.6)	1,613 (1,600-1,626)	1 in 7	166 (0.1)	5 (4-5)	1 in 2,197
**Females**							
**Total**	5,782,147	2,238,302 (38.7)	1,027 (1,026-1,029)	1 in 10	747,498 (12.9)	327 (326-328)	1 in 31
25.0–29.9	2,584,807	910,583 (35.2)	900 (899-902)	1 in 12	673,371 (26.1)	663 (662-665)	1 in 16
30.0–34.9	1,641,270	643,529 (39.2)	1,047 (1,044-1,049)	1 in 10	59,839 (3.6)	98 (97-99)	1 in 103
35.0–39.9	863,186	359,870 (41.7)	1,174 (1,170-1,178)	1 in 9	10,261 (1.2)	34 (33-35)	1 in 296
40.0–44.9	409,328	182,244 (44.5)	1,327 (1,321-1,333)	1 in 8	3,012 (0.7)	22 (21-23)	1 in 450
≥45.0	283,556	142,076 (50.1)	1,614 (1,606-1,623)	1 in 7	1,015 (0.4)	12 (11-12)	1 in 852

[a] The subset excluded 2,261,509 (16.9%) individuals with evidence of any of the following during the study period: eating disorders (n=14,523 [0.1%]), antiobesity medication prescriptions (n= 719,633 [5.4%]), bariatric surgery (n=56,210 [0.4%]), malignancy (n=469,325 [3.5%]), metastatic solid tumor (n=115,177 [0.9%]), Charlson comorbidity index≥3 (n=1,401,746 [10.5%]), chemotherapy(n=28,574 [0.2%]), end-of-life care (n=71,890 [0.5%]), hospice care (n=2,304 [0.0%]).

[b]Model 1 is a Poisson model on the sample of US adults with BMI≥25, with the outcome of a ≥5% reduction in initial weight, exposure in person-years, and the following covariates: two-way interaction between initial BMI category and sex, including main effects, and initial age group (17–19, 20–29, 30–39, 40–49, 50–59, 60–69, 70+).

© 2023 Kompaniyets L et al. *JAMA Network Open.*
