# Supplementary material for: Probability of 5% or Greater Weight Loss or BMI Reduction to Healthy Weight Among Adults With Overweight or Obesity
Source: JAMA Netw Open. 2023 Aug 7;6(8):e2327358. doi: 10.1001/jamanetworkopen.2023.27358 (PMC10407685; doi:10.1001/jamanetworkopen.2023.27358)
Supplement: Supplement 2. — Data Sharing Statement [file jamanetwopen-e2327358-s002.pdf]

Data Sharing Statement

Kompaniyets. Probability of 5% or Greater Weight Loss or BMI Reduction to Healthy Weight Among Adults With Overweight or Obesity. *JAMA Netw Open*. Published August 07, 2023. doi:10.1001/jamanetworkopen.2023.27358

Data
Data available: No
